# Supplementary figures and images for: m6A minimally impacts the structure, dynamics, and Rev ARM binding properties of HIV-1 RRE stem IIB
Source: PLoS One. 2019 Dec 11;14(12):e0224850. doi: 10.1371/journal.pone.0224850 (PMC6905585; doi:10.1371/journal.pone.0224850)

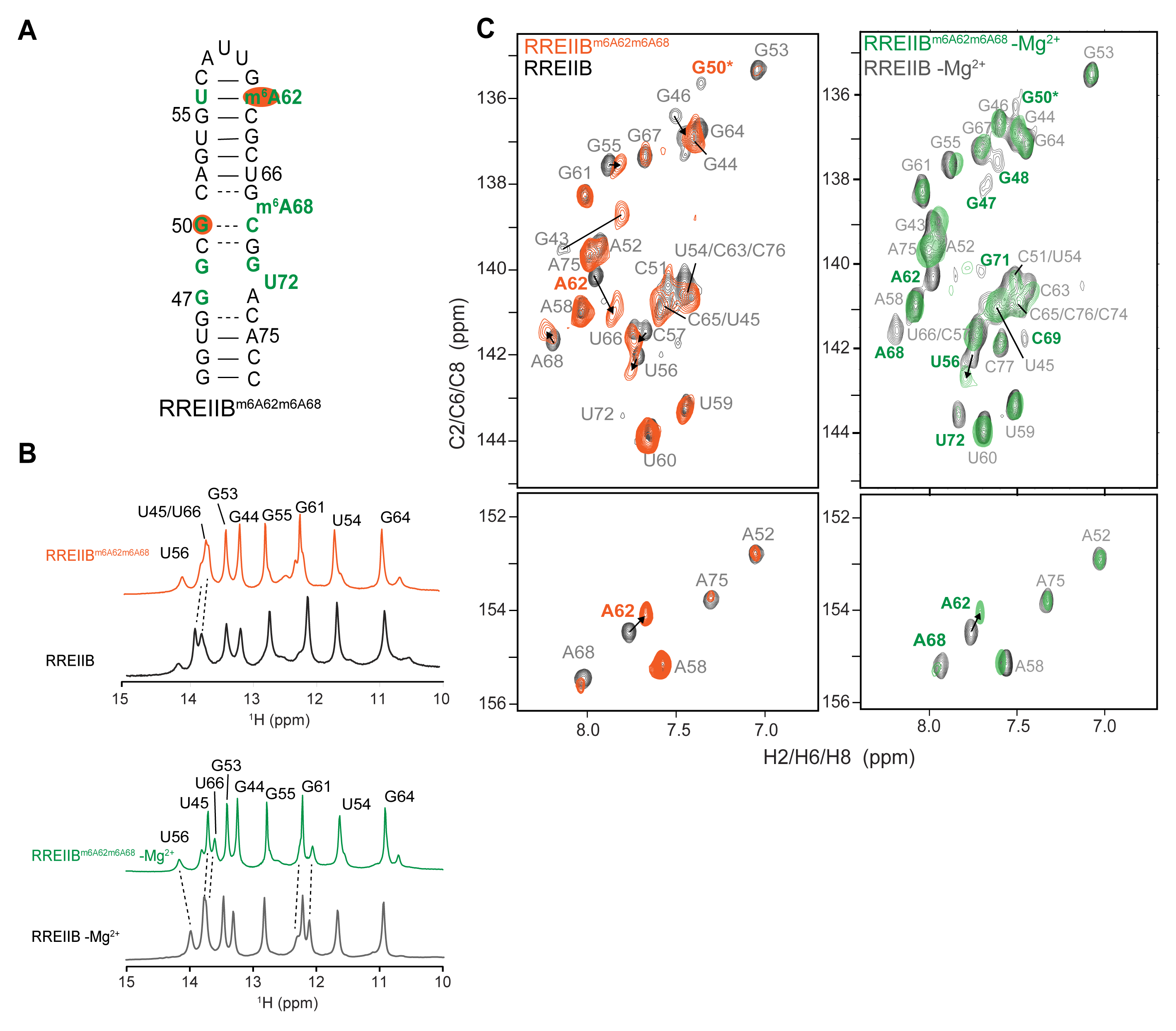

Supplement: S1 Fig — (A) Secondary structure of RRE2Bm6A62,68. Resonances exhibiting line-broadening and perturbations in 2D [1H, 13C] aromatic HSQC spectra are shown in orange (with Mg2+) and green (no Mg2+), respectively. The comparison of 1D imino spectra (B) and 2D [1H,13C]-HSQC spectra (C) of RREIIBm6A62m6A68 and RREIIB in the presence (orange) and absence (green) of 3 mM Mg2+. Arrows indicate chemical shift perturbations while ambiguous assignments are denoted using an asterisk. (TIF) [file pone.0224850.s001.tif]

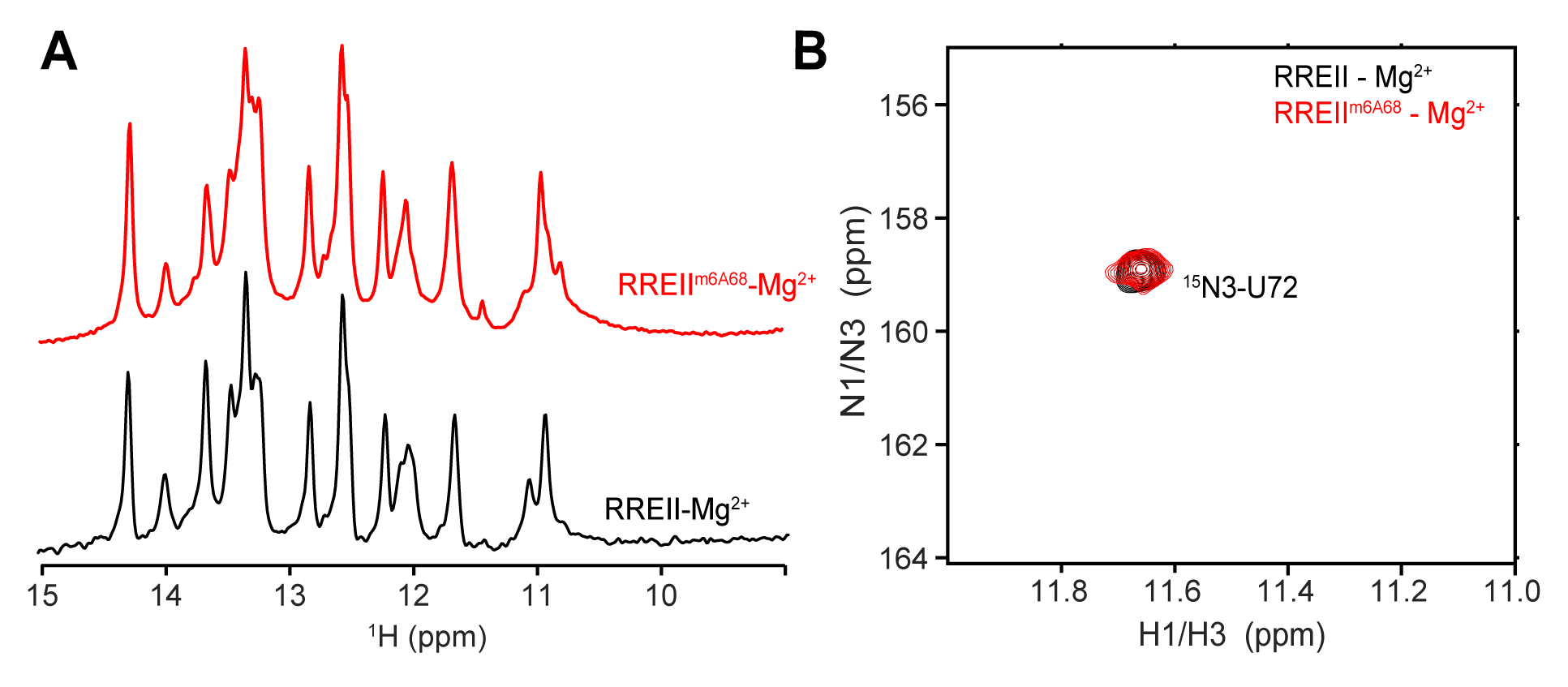

Supplement: S2 Fig — (A) The comparison of 1D imino spectrum of RREIIm6A68 and RREII without Mg2+. (B) 2D [1H,15N]-HSQC spectra of site-specifically labeled 15N3-U72-RREIIm6A68 and 15N3-U72-RREII without Mg2+ at 25°C showing a single imino resonance at the characteristic chemical shift region (~11.6 ppm) expected for a G-U bp in ES1 and ES2. The sample conditions were 0.3–0.5 mM RNA in 15 mM sodium phosphate, 25 mM NaCl, 0.1 mM EDTA at pH 6.4. (TIF) [file pone.0224850.s002.tif]
